# Expanding the genotype–phenotype spectrum in hereditary colorectal cancer by gene panel testing

**DOI:** 10.1007/s10689-016-9934-0

**Published:** 2016-09-30

**Authors:** Anna Rohlin, Eva Rambech, Anders Kvist, Therese Törngren, Frida Eiengård, Ulf Lundstam, Theofanis Zagoras, Samuel Gebre-Medhin, Åke Borg, Jan Björk, Mef Nilbert, Margareta Nordling

**Affiliations:** 1 Department of Molecular and Clinical Genetics, Institute of Biomedicine, Sahlgrenska Academy at University of Gothenburg, Gothenburg, Sweden; 2 Division of Oncology and Pathology Department of Clinical Sciences Lund, Lund University, Medicon Village, 22381 Lund, Sweden; 3 Department of Surgery, Sahlgrenska Academy at University of Gothenburg, Sahlgrenska University Hospital/Östra, 416 85 Gothenburg, Sweden; 4 Division of Clinical Genetics, Department of Laboratory Medicine, Lund University, Lund, Sweden; 5 Department of Clinical Genetics, Office for Medical Services, Division of Laboratory Medicine, Lund, Sweden; 6 The Swedish Polyposis Registry, Department of Medicine, Karolinska Institute, Stockholm, Sweden; 7 The HNPCC-register, Hvidovre University Hospital, Copenhagen University, Hvidovre, Denmark

**Keywords:** Colorectal cancer, FAP, Familial adenomatous polyposis, Gene panel, Hereditary, Colorectal cancer, Lynch syndrome

## Abstract

Hereditary syndromes causing colorectal cancer include both polyposis and non-polyposis syndromes. Overlapping phenotypes between the syndromes have been recognized and this make targeted molecular testing for single genes less favorable, instead there is a gaining interest for multi-gene panel-based approaches detecting both SNVs, indels and CNVs in the same assay. We applied a panel including 19 CRC susceptibility genes to 91 individuals of six phenotypic subgroups. Targeted NGS-based sequencing of the whole gene regions including introns of the 19 genes was used. The individuals had a family history of CRC or had a phenotype consistent with a known CRC syndrome. The purpose of the study was to demonstrate the diagnostic difficulties linked to genotype-phenotype diversity and the benefits of using a gene panel. Pathogenicity classification was carried out on 46 detected variants. In total we detected sixteen pathogenic or likely pathogenic variants and 30 variants of unknown clinical significance. Four of the pathogenic or likely pathogenic variants were found in *BMPR1A* in patients with unexplained familial adenomatous polyposis or atypical adenomatous polyposis, which extends the genotype-phenotype spectrum for this gene. Nine patients had more than one variant remaining after the filtration, including three with truncating mutations in *BMPR1A*, *PMS2* and *AXIN2*. CNVs were found in three patients, in upstream regions of *SMAD4*, *MSH3* and *CTNNB1*, and one additional individual harbored a 24.2 kb duplication in *CDH1* intron1.

**Electronic supplementary material:**

The online version of this article (doi:10.1007/s10689-016-9934-0) contains supplementary material, which is available to authorized users.

## Introduction

Around 6 % of colorectal cancers (CRC) comprise hereditary syndromes for which high-penetrant mutations are found in syndrome-specific genes [37]. Lynch syndrome, familial adenomatous polyposis (FAP), MUTYH associated polyposis (MAP), Juvenile Polyposis syndrome (JPS), PTEN hamartoma tumor syndrome and Peutz–Jeghers syndrome (PJS) are among the most well known.

The introduction of next generation sequencing (NGS) using whole-genome sequencing (WGS), whole-exome sequencing (WES) and multigene panels have made it possible to identify a spectrum of new mutations and also new causative genes in hereditary CRC. New syndromes have been described like the recently reported, Polymerase Proofreading-Associated Polyposis (PPAP) and *NTHL1*-associated polyposis [31, 50]. Established syndromes with unsolved causative genetic mechanisms are also gradually being explored, which is the case for the hereditary mixed polyposis syndrome (HMPS) [12, 51].

CRC syndromes have historically been defined based on family history and/or genetics as well as tumor characteristics. For hereditary non-polyposis colorectal cancer, Amsterdam criteria, tumor testing for microsatellite instability (MSI) as well as presence of causative mutations in the mis-match repair (MMR) genes, have been used for sub-classification [19]. Overlaps in mutation spectrum between polyposis and non-polyposis syndromes are also recognized. The HMPS is characterized by the presence of polyps of several histological types localized to the large bowel. Adenomatous polyps as well as polyps of serrated or sessile serrated type can be present. The JPS and HMPS may show overlapping phenotypes and may appear indistinguishable [13, 26]. In HMPS, duplications in the regulatory domain of *GREM1* were recently identified [12, 34], but except from the *GREM1* regulatory pathogenic duplications, also causative mutations in *BMPR1A* have been found [4]. Mutations in *BMPR1A* have also been reported in hereditary non-polyposis CRC with microsatellite stable (MSS) tumors (FCC type-X) [8, 29]. The duplication upstream *GREM1* in a family with a few polyps of a more juvenile histology reported by our group, demonstrates the complexity of the phenotype-based classification of this syndrome [34].

Multigene panel testing in Lynch syndrome has recently been used to identify mutations in unexpected high penetrant cancer-predisposing genes (e.g. *BRCA1* and *BRCA2*) [45, 54]. Several studies have also demonstrated cost benefits as well as gain in mutation detection when using multigene panels compared to analyses of single- or a few genes [9, 39, 40, 45].

Additional research is required to understand the genetic heterogeneity in these groups and the diversity in genotype to phenotype correlations. In our study we used a multigene CRC-panel consisting of 19 high risk- and moderate risk genes as well as clinically less well defined genes in the MMR system and the *wnt* signaling pathway [28, 48]. The panel was applied to patients diagnosed with CRC divided into six clinical subgroups. The classification into subgroups was based on family history and/or phenotype of the disease. All patients had initially been referred for a specific diagnostic test, Lynch syndrome or a polyposis syndrome. The purpose of this study was to demonstrate the diagnostic difficulties associated with genotype to phenotype diversity. In the performed study, which also included screening for large deletions and duplications, we were able to demonstrate improved mutation detection frequencies compared to conventional multi-step analyses. The strategy also allowed for reduction in costs compared to previously used screening procedures.

## Materials and methods

Ninety-one index patients were included in this study. Clinical characteristics of the index patients and families are summarized in Supplementary Table 1. The medical journals were reviewed and the patients were divided into six clinical subgroups. All the patients were originally referred for clinical FAP- and/or Lynch syndrome mutation analyses during 2000–2015, but no mutations were identified in the genes analyzed. The subgroups were: (1**)** CRC familial or unknown inheritance, not polyposis, (2**)** unexplained adenomatous polyposis >100 polyps, inheritance, (3**)** unexplained adenomatous polyposis 1–100 polyps, inheritance, (4**)** Unexplained adenomatous polyposis, unknown inheritance, (5**)** familial or simplex atypical polyposis/mixed polyposis/serrated polyposis and (6**)** polymerase proofreading associated polyposis (PPAP). The study has been approved by the local ethics committee at the University of Gothenburg, Sweden.

### DNA extraction, amplification and sanger-sequencing

Genomic DNA was extracted using the BioRobot EZ1 (Qiagen, Hilden, Germany) with the EZ1 DNA Blood 350 µl kit (Qiagen). Amplification, purification and Sanger sequencing were carried out as described previously [14]. Primers used for direct sequencing were identical to those used in the amplification reactions. All primer information is available upon request. All variants found by capture NGS (Next Generation Sequencing) were confirmed with Sanger sequencing.

### Library preparation, hybridization capture and MPS sequencing

DNA samples were quantified using the Qubit system (Life Technologies, Carlsbad, CA, USA). Two µg of DNA were fragmented using the Covaris S2 Ultrasonicator (Covaris, Woburn, MA, USA), the samples were then analyzed on the Bioanalyzer (Agilent Technology, Santa Clara, CA, USA) for correct fragment sizes. The SureselectXT Custom 3-5.9 Mb library kit (Agilent Technology, Santa Clara, CA, USA) was used for the capture and included 19 genes *APC, MUTYH, BMPR1A, SMAD4, STK11, PTEN, MLH1, MSH2, MSH6, PMS2, EPCAM, CDH1* were all high risk genes. *MLH3, MSH3, PMS1,AXIN2, CTNNB1,CHEK2* and *MET* were genes that are part of the MMR system, *wnt* signaling and/or were found at the time of the development of the test to have an increased but not well defined risk [24, 28, 44, 53]. Regions of 50 kb upstream and downstream of all genes and all intronic regions were included in the target region. For the *APC* gene an additional region of 100 kb upstream was included, since causative deletions in the promoter region have been found [23, 32, 35, 41]. For the *MET* gene only coding exons were targeted. Eight samples were pooled before capture and the concentration of each pooled library was determined by using the Qubit and the Bioanalyzer. Sequencing was performed on the Illumina HiSeq 2000 (Illumina, San Diego, CA, USA) with 2 × 94 or 2 × 97 bp paired end reads.

### Analysis of sequencing data

An in-house analysis pipeline was used in which the main steps after demultiplexing included read alignment to the reference human genome hs37d5ss (1000 genome with decoy sequences) by Novoalign, marking of PCR duplicates (Picard tools, http://picard.sourceforge.net) and quality score recalibration, indel realignment and variant calling performed with the Genome Analysis Tool Kit (GATK) package [27]. For all samples and positive controls variants were called with GATK UnifiedGenotyper with a call confidence of 10.

### Copy number variation (CNV) analysis

The CNV analysis was based on read depth, one read-pair represents one data point in a sliding window over the target region. A normalized coverage depth ratio including GC-normalization between a sample and an average of 23 normal samples (baseline) were computed. Detection of abnormal coverage ratios were found by visual inspection of plots of the coverage ratios over the targeted regions. Deletions were detected as a lower coverage (cut off 0.75) and duplication as a higher coverage (cut off 1.25). Regions with an abnormal coverage ratio were further inspected in IGV (Integrative Genomics Viewer) and breakpoints were analyzed [33].

### Filtration of variants and databases

Variants in exons and in ± 20 bp flanking intronic sequences were evaluated for pathogenicity. Truncating nonsense, frameshift indels and variants located in consensus splice-acceptor and-donor sites were presumed and evaluated as disease causing. All other variants, synonymous and non-synonymous, were compared with the following public databases; the Single Nucleotide Polymorphism database (dbSNP) together with 1000 Genomes [1], the National Heart, Lung and Blood Institute (NHLBI) Exome Sequencing Project (ESP) (http://evs.gs.washington.edu/EVS/), ExAc (Exome Aggregation Consortium, Cambridge, MA (URL: http://exac.broadinstitute.org) [20 (02, 2014) accessed]), TCGA data (www.cbioportal.org) and with in-house information. Variants with a minor allele frequency (MAF) of ≤1 % were further analyzed, the rest of the variants were treated as polymorphisms, this also included likely benign variants. Thirty-two missense variants with an MAF ≤1 % were classified based on three in silico protein prediction tools, SIFT, PolyPhen-2 and CADD. SIFT (Sorting Intolerant From Tolerant) predicts a damaging mutation if the score is ≤0.05, and tolerated if the score is >0.05 [20]. PolyPhen-2 (Polymorphism Phenotyping version 2), predicts probably damaging and possibly damaging mutations with a higher confidence if values are near 1 [3]. The Combined Annotation Dependent Depletion (CADD) is a method to measure deleteriousness by comparing the annotation of fixed or almost fixed derived alleles with those of simulated variants. Several parameters are taken into account when using CADD, including, allelic diversity, annotation and functionality, pathogenicity, disease severity, experimentally measured regulatory effects, complex trait associations and highly ranked known pathogenic variants within individual genomes. Variants that are more likely to be observed in the genome are proposed to be more benign while variants that are more likely to be simulated (not observed) are proposed to have a more deleterious effect. This is measured in a Phred-like scale C-score, were a score of 10 represents the 10 % most deleterious substitutions that can be done to the human genome and a score of 20 represents the 1 % most deleterious variants. Higher score is associated with a higher probability of a deleterious effect with a recommended cut-off at 15 [16].

Classification of variants by the InSiGHT database [46] was considered correct. For variants not included in this database published literature and classification done by HGMD [42] as well as Leiden open source variation (LOVD) databases and also ClinVar [22] were used in combination with in-house information to make a manual classification. The manual classification criteria was used according to the five-class system following guidelines from the International Agency for Research on Cancer (IARC): 1 = Benign, 2 = Likely benign, 3 = Variant of Unknown clinical Significance (VUS), 4 = Likely pathogenic and 5 = Pathogenic.

## Results

The gene panel was applied to 91 patients, previously tested negative for mutations in the polyposis genes (*APC, MUTYH, BMPR1A, SMAD4, STK11*) and/or a combination of different MMR genes (*MLH1, MSH2, MSH6, PMS2,*) depending on the primary indication when the referral was issued. The patients were sub-grouped based on their clinical characteristics (Supplementary Table 1). Sequencing was performed over the entire gene regions as described and all coding regions were covered at least 30 × except for *CDH1* ex1, *EPCAM* ex1, *MSH3*, ex1 and *MLH1* ex12 which in five samples were covered at least 25x. For the whole targeted region the mean coverage was 417x in all 91 samples. The analyses of variants included the coding region and ± 20 bp of intronic sequences. The CNV analysis was based on the entire covered gene regions.

In total 8 pathogenic class 5 and 8 likely pathogenic class 4 variants were found (Tables 1, 2). This gives a mutation detection frequency of 8.8 % (8/91) for the class 5 variants only and a frequency of 17.6 % (16/91) when also class 4 variants are included. These results are in concordance with the results obtained in other studies of similar gene panels [6, 18]. Two pathogenic variants in *PMS2* in patients I:26 and I:50 were missed in the initial analyses of the MMR genes performed in an external laboratory. Thirty-two missense variants, all of them found in a heterozygote state, with MAF ≤1 %, according to the filtration criteria, were analyzed and classified manually or according to the InSiGHT database [46] in the case the variant was included in this database. The results are presented in Table 2 and include four class 5 pathogenic variants, two likely pathogenic class 4 variants and 26 class 3 variants of unknown clinical significance (VUS). The *APC* variant, c.1902 T > G, was recently found to have a major splicing effect on exon 14 resulting in loss of this exon [10]. The variant was found in a patient (III:61) with unexplained familial adenomatous polyposis (1–100 polyps), this patient also had a VUS, *APC* c.4472T > A, p.Phe1491Tyr. Both of these variants segregated with affected individuals and neither of them were found among healthy individuals from the family. Two class 5 variants were found in *MUTYH,* one each in patients III:71 and I:42, respectively. The c.536A > G, p.Tyr179Cys and c.1187G > A, p.Gly396Asp were found in a heterozygote state and are classified as pathogenic if found homozygote or in a compound heterozygote state.

**Table 1:** Truncating variants among 91 index patients

Clinical group: patient number	Gene	Genomic position (GRCh37/hg19)/dbSNP (rs)	Genotype	Coding effect	Protein variant	ExAc All^#^	Classification^
I:32	SMAD4	chr18:g. 48591947-4859195951del	NM_005359: c.1110_1114del	Frameshift	p.His371Aspfs*5	Not present	5
I:20	CHEK2	chr22: g.29130389	NM_007194: c.319 + 2T > A	Splicing	p.?	Not present	4
I:26	PMS2	chr7: g.6026808	NM_000535: c.1588C > T	Nonsense	p.Gln530*	0.0017 %	4
I:50	PMS2	Chr7:g.6035204_6035207del	NM_000535: c.861_864del	Frameshift	p.Arg287Serfs*19	0.0017 %	5^in^
I:91	PMS2	Chr7:g.6031690	NM_000535:c.904-2A > G	Splicing	p.?	Not present	4
IV:69	MLH3	chr14: g.75506621/rs193219754	NM_001040108: c.3563 C > G^ho^	Nonsense	p.Ser1188*	0.019 %	4
I:55	AXIN2	Chr17:g.63554485del	NM_004655.3:c.254del	Frameshift	p.Leu85Tyrfs*24	Not present	4
II:59	BMPR1A	chr10: g.88659789	NM_004329: c.441delT	Frameshift	Phe147Leufs*18	0.00082 %	5
IV:76	BMPR1A	chr10: g.88649983	NM_004329: c.230 + 2T > C	Splicing	p.?	Not present	4
V:87	BMPR1A	chr10: g.88679023	NM_004329: c.969delT	Frameshift	p.Cys323Trpfs*41	not present	5

^^^, manual classification unless indicated with “in”, when marked with “in” classified according to InSiGHT [46], ho, this patient was homozygote for the variant; ^#^, URL: http://exac.broadinstitute.org

**Table 2:** Classification of missense variants with a MAF < 1 % among 91 index patients

Clinical group: patient number	Gene	Position (GRCh37/hg19)/dbSNP (rs)	Genotype	Consequence	ExAc All^#^	SIFT^&^	PolyPhen-2^¤^	C-score^+^	Classification^
III:61	APC	Chr5:112170806	NM_000038:c.1902T > G	Exon 14 skipping	Not present	Deleterious	Probably damaging	20.9	5
III:61	APC	Chr5:112175763	NM_000038:c.4472T > A	p.Phe1491Tyr	Not present	Deleterious	Probably damaging	22.8	3
V:89	APC	Chr5:112176431/rs148275069	NM_000038:c.5140G > A	p.Asp1714Asn	0.020 %	Deleterious	Benign	13.5	3
I:1	APC	Chr5:112177415	NM_000038:c.6124T > C	p.Cys2042Arg	Not present	Deleterious	Probably damaging	15.7	3
I:38	APC	Chr5:112176196/rs137988845	NM_000038:c.4905G > A	p.Gly1635Gly	0.038 %	Not ava	na	3.2	3
I:50	APC	Chr5:112178693	NM_000038:c.7402T > C	p.Ser2468Pro	0.002 %	Tolerated	Possibly damaging	23.5	3
I:11	AXIN2	Chr17:63532528/rs138287857	NM_004655:c.2051C > T	p.Ala684Val	0.19 %	Deleterious	Possibly damaging	22.6	3
II:58	BMPR1A	Chr10:88683199/rs199476089	NM_004329:c.1409T > C	p.Met470Thr	Not present	Deleterious	Probably damaging	21.6	4
V:90	CDH1	Chr16:68855966/rs35187787	NM_004360:c.1774G > A	p.Ala592Pro	0.32 %	Deleterious	Benign	15.3	3
V:87	CHEK2	Chr22:29130520/rs141568342	NM_007194:c.190G > A	p.Glu64Lys	0.016 %	Tolerated	Possibly damaging	18.2	3
V:87	CHEK2	Chr22:29121087/rs17879961	NM_007194:c.470T > C	p.Ile157Thr	0.41 %	Deleterious	Probably damaging	21.1	4
III:27	CTNNB1	Chr3:41275730	NM_001904:c.1625G > A	p.Arg542His	0.00082 %	Deleterious	Benign	18	3
I:1	MET	Chr7:116340039/rs201687037	NM_001127500:c.901A > G	p.Thr301Ala	0.022 %	Tolerated	Probably damaging	11.3	3
I:57, IV:79	MLH1	Chr3:37089130/rs35001569	NM_000249:c.1852A > G	p.Lys618Glu	0.34 %	Deleterious	Probably damaging	27.8	3^in^
IV:85	MLH1	Chr3:37090050/rs63750109	NM_000249:c.1939G > A	p.Val647Met	0.015 %	Deleterious	Benign	18	3^in^
I:41	MLH1	Chr3:g.37053590/rs63751711	NM_000249:c.677G > A	ex 8 skipping	Not present	Deleterious	Probably damaging	34	5^in^
I:8, I:10, I:47	MLH3	Chr:75514489/rs28756986	NM_001040108:c.1870G > C	p.Glu624Gln	0.73 %	Tolerated	Probably damaging	17	3
I:4, I:33	MLH3	Chr14:75509146/rs28757008	NM_001040108:c.3315C > A	p.Asp1105Glu	0.29 %	Tolerated	Possible damaging	17.7	3
II:24	MLH3	Chr14:75506718/rs184741686	NM_001040108:c.3466G > A	p.Val1156Ile	0.011 %	Tolerated	Benign	16.8	3
IV:81	MLH3	Chr14:75483796/rs28939071	NM_001040108:c.4351G > A	p.Glu1451Lys	0.074 %	Tolerated	Probably damaging	14.	3
I:12, I:37	MSH3	Chr5:80109479/rs41545019	NM_002439:c.2732T > G	p.Leu911Trp	0.24 %	Deleterious	Probably damaging	17.4	3
I:55	MSH2	Chr2:g.47657079/rs63751650	NM_000251.2:c.1275A > G	p.Glu425Glu	0.01 %	na	na	13.6	3^in^
I:92	MSH2	Chr2:g.47703513/rs587779127	NM_000251.2:c.2013T > A	p.Asn671Lys	Not present	Deleterious	Probably damaging	28.2	3^in^
IV:79	MSH6	Chr2:48027790	NM_000179:c.2668G > T	p.Val890Phe	Not present	Deleterious	Possibly damaging	12.9	3
I:56	MSH6	Chr2:g.48030612	NM_000179:c.3226C > T	p.Arg1076Cys	0.0091 %	Deleterious	Probably damaging	32	3^in^
III:71	MUTYH	Chr1:45798475/rs34612342	NM_001128425:c.536A > G	p.Tyr179Cys	0.16 %	Deleterious	Probably damaging	19	5^ho^
I:42	MUTYH	Chr1:g.45797228/rs36053993	NM_001128425:c.1187G > A	p.Gly396Asp	0.28 %	Deleterious	Probably damaging	29.4	5^ho^
I:51	MUTYH	Chr1:g.45797846/rs138089183	NM_001128425:c.925C > T	p.Arg309Cys	0.044 %	Tolerated	Benign	13.9	3^ho^
I:33	PMS1	Chr2:190660640	NM_000534:c.278G > A	p.Arg93His	0.0025 %	Deleterious	Probably damaging	36	3
IV:31	PMS1	Chr2:190708701/rs143010673	NM_000534:c.594G > T	p.Trp198Cys	0.015 %	Deleterious	Probably damaging	20.8	3
I:51	PMS1	Chr2:g.190660536/rs143323454	NM_000534:c.174G > T	p.Gly58Gly	0.33 %	na	na	9.3	3
I:48	STK11	Chr19:g.1226474/rs199973552	NM_000455:c.1130C > T	p.Ala377Val	0.01 %	Tolerated	Benign	13.7	3

^#^URL: http://exac.broadinstitute.org; and [20]; ¤ [3]; ho, pathogenic in homozygote state;^ ^^, manual classification unless indicated with in, when marked with in classified according to InSiGHT [46], na, not available

Nine patients had more than one variant remaining after the filtration, including three with truncating variants in *BMPR1A*, *PMS2* and *AXIN2*. The *BMPR1A* c.969delT variant (Table 1) was found together with one likely pathogenic variant (class 4) in *CHEK2* c.470T > C, p.Ille157Thr (Table 2), in an mixed polyposis case (V:87), additionally this patient carried a *CHEK2* VUS, c.190G > A, p.Glu64Lys (Table 2). A truncating variant in *PMS2* c.861_864del (Table 1) was found together with the VUS *APC* c.7402T > C, p.Ser2468Pro (Table 2) in patient I:50. The *AXIN2* c.254del (Table 1) variant and the synonymous VUS *MSH2* c.1275A > G (Table 2) were both found in patient I:55.

Tumor characteristics e.g. MSI and IHC can be of value for interpretation of the VUS. For 52 of these patients we had results from only MSI tests or for both MSI and IHC tests (Supplementary Table 1).When investigating the VUS present among these patients there are some findings. Patient I:47 has a tumor which is MSI-H and present a loss of MLH1/PMS2 proteins, this patient has a VUS in *MLH3,* c.1870G > C, p.Glu624Gln. This VUS was also found in two patients with an MSS (I:8, I:10) tumour phenotype. The variant is interpreted differently between the in silico protein predication tools used, it has a low CADD score (17) and is quite common in the ExAc population database (0.73 %). Since tumors from patients with this variant can be both MSI or MSS, it is difficult to conclude the pathogenic effect of the variant. In two patients with a MSI-H tumour phenotype, one *MSH6*:c.3226C > T, p.Arg1076Cys (I:56) and one *MSH2*:c.2013T > A, p.Asn671Lys (I:92) VUS were found. These variants are predicted damaging by all in silico protein predication tools, exhibit a high CADD score (32 respectively 28.2) and are rare (0.0091 %) or not present in the population database ExAc. Both variants might be predicted to have a likely pathogenic effect. TCGA data (www.cbioportal.org) shows a high functional impact for the *MSH6* variant. In the patients with an MSS tumor phenotype, eight unique MMR variants were found. The variants exhibit conflicting in silico protein predication results. Combined with a lower CADD score in general, the variants might be predicted to have a likely benign effect, consistent with their MSS phenotype.

Four structural variants were found and they are presented in Table 3. An individual (patient III:65) from a family with phenotypic AFAP was found to carry a 1.9 kb heterozygote deletion located 2 kb upstream of *SMAD4* (hg19/chr18:g.48537165_48539080del). The deleted region includes an insulator element 200 bp in size (chr18:g.48537803-48538002). Additional upstream deletions were found in *MSH3* (I:34) and *CTNNB1* (I:57). Another patient (I:6) had a 24.2 kb duplication in *CDH1* intron 1 (hg19/Chr16:g.68802080_68826280del).

**Table 3:** Structural variations detected among 91 index patients

Clinical group: patient number	Location	Genomic position (GRCh37/hg19)/dbSNP (rs)	Approximate size (kb)	Classification*
III:65	Upstream SMAD4	hg19/chr18:g.48537165_48539080del	1.9	3
I:6	Intron 1 CDH1	hg19/Chr16:g.68802080_68826280dup	24.2	3
I:34	Upstream MSH3	hg19/Chr5:g.79902126_79904625del	2.5	3
I:57	Upstream CTNNB1	hg19/Chr3:g.41200986_41203204del	2.2	3

* manual classification

### Discussion

In this study we show the importance of using multigene panels which allows for a parallel comprehensive screening for CRC syndromes. Mutations in *BMPR1A* have been found in an extended phenotypic spectrum beyond juvenile polyposis, including HMPS, AFAP simplex, familial colorectal cancer type X (FCCX) and early onset CRC without familial history and MSI negative tumours [4, 8, 29, 30]. To this spectrum we add a patient with an atypical polyposis (V:87, this patient also carries two *CHEK2* variants, Table 2) and three patient with unexplained adenomatous polyposis and different number of polyps. Patient IV:76 had a splice-site variant c.230 + 2T > C (class 4), II:59 had a truncating variant, c.441delT, Phe147Leufs*18 (class 5) and the last patient (II:58) had a probable pathogenic (class 4) missense mutation, c.1409 C > T, p.Met470Thr in *BMPR1A*. This missense mutation has previously been found in a patient with a juvenile polyposis phenotype and around 300 polyps throughout the entire gastrointestinal tract [15]. Two patients from Group I, “CRC familial or unknown inheritance not polyposis”, had variants in *AXIN2*. In one of these patient with late onset of CRC a truncating *AXIN2* variant was found together with an *MSH2* variant of unknown significance (I:55). The second patient (I:11) presented with an *AXIN2* missense variant c.2051C > T, p.Ala684Val. Variants in *AXIN2* have been reported in patients with CRC and oligodentia and in patients with oligodentia solely [21, 52]. It is suggested that truncating pathogenic variants in *AXIN2* are more likely to predispose carriers to syndromic oligodontia and colorectal cancer compared to missense variants [25]. To our knowledge oligodonita was not present in any of our patients.

We found a large deletion in the regulatory region of *SMAD4* in a patient with unexplained adenomatous polyposis (1–100 polyps) (III:65). An insulator element that may act as a barrier to enhancer action is located in the deleted region. Transcription of genes beyond the insulator is not stimulated by the enhancer when the insulator is active. This deletion might therefore have an effect on the expression of the gene. In a recent study two *SMAD4* mutations in patients without juvenile polyps were identified, one with around 20–99 adenomatous polyps and the other one without reported polyps, which further extends the phenotypical spectrum for this gene [6].

There is a complexity of combinations of possible ligand receptors and downstream effectors in the BMP/TGFR-β signalling pathways and this might explain part of the genotype-phenotype relationship. There might also be a genotype-phenotype correlation depending on where in the gene the mutation is located. Several genes in the BMP/TGFR-β signalling pathway are mutated in hereditary CRC as well as sporadic CRC and possibly inactivation of also other genes in this pathway might predispose carriers to CRC. It seems as if patients with mutations in *APC*, *BMPR1A*, *SMAD4* and *GREM1* can have similar polyposis phenotypes but carriers of *GREM1* mutation with HMPS might not have the same risk for extra-colonic disease as patients with *BMPR1A* mutations and HMPS [47].

In a recent multigene-panel based CRC study 1.4 % (8/586) had *CHEK2* risk alleles or truncating mutations, two of the patients had the c.470T > C, p.Ile157Thr, variant and four c.1100delC alleles, all had polyps or CRC, none of them had a personal history of breast cancer, but six had at least one family member with breast cancer [6]. Around 2 % (2/91) of our patients had *CHEK2* variants, V:87 had both c.190G > A, p.Glu64Lys and c.470T > C, p.Ile157Thr and I:20 had the splice variant c.319 + 2T > A, and they did present with polyps or CRC but no breast cancer has been reported in the families as we know of. Variants in *CHEK2* still remain of uncertain clinical relevance as is further emphasized by the fact that V:87 also carried a truncating probably pathogenic variant in the *BMPR1A* gene (c.969delT). In a recent study *CHEK2* variants have been found among individuals with various types of cancer, which might be partly due to the high population frequency of the common *CHEK2* variants (c.1100delC and p.Ile157Thr) [45].

The truncating *MLH3* mutation c.3563 C > G, p.Ser1188* was found in homozygote state in an unexplained polyposis case (IV:69) with duodenal polyps and CRC. The MLH3 protein as well as the PMS1 protein can dimerize with MLH1 and assist in single nucleotide mis-match DNA-repair, but their roles are not well understood [38]. Variants in the genes have been found in patients without a family history, in some cases also in sporadic patients and/or in healthy controls. Variants have also been found together with other MMR gene variants, suggesting *PMS1* and *MLH3* to be low risk genes in Lynch syndrome [17]. The clinical significance of the variant we report here, is therefore difficult to estimate. However, recently compound heterozygote loss of function (LoF) germline mutations in the *MSH3* gene were identified in patients with an unexplained adenomatous polyposis. The data presented by Adam et al. strongly support disease causing *MSH3* mutations to follow a recessive mode of inheritance [2]. A comparable scenario might possibly also be considered for mutations in *MLH3*.

When comparing the VUS in the MMR genes to the corresponding results from the MSI and IHC test of the tumours, some conclusion might be drawn concerning the pathogenicity. Two variants, one in *MSH6* c.3226C > T, p.Arg1076Cys (I:56) and one in *MSH2* c.2013T > A, p.Asn671Lys (I:92), that were identified in patients who presented with a MSI-H phenotype (no IHC results were available), might be predicted to be likely pathogenic. Both of these variants are predicted damaging by all the protein predication tools used, they also have a very high CADD score and are very rare or not present in the population database ExAc. TCGA data shows a high functional impact for the *MSH6* variant. It is feasible to predict these variants as presumably likely pathogenic at this point until more functional data is available.

The patient (I:6) with the intronic duplication in *CDH1*also had breast cancer. It is known that *CDH1* mutations can be found in patients with lobular breast cancer and in hereditary diffuse gastric cancer. Although no obvious functional elements are found in this region it cannot be ruled out that the duplication has an effect on the transcription or regulation of the gene.

The search for germ-line mutations in risk individuals have been focused on mutations associated with highly penetrant disease phenotypes, which include a stepwise approach leading to an expensive strategy and underestimation of familial cases [43]. The increased use of multigene panels have already shown a higher mutation detection rate compared with traditional testing based on clinical criteria [6, 18], as is also confirmed by this study. The reason for this is probably a large genetic heterogeneity and overlapping clinical presentation of the different CRC syndromes. Limited knowledge of the medical and/or family history or an atypical presentation of the CRC syndromes might lead to an incorrect diagnosis of patients. The possibility of panel-based testing is beneficial not only for the patient but also for time and cost savings. However, there is also a complexity of information that can result from a multigene-panel test. Variants may also be coincidental or explain only part of the clinical phenotype. Segregation analyses could in these cases be used to further understand the clinical significance of variants. In this study, when also structural variants are included, in total 33 % (30/91) of the patients have at least one VUS. When eliminating those with a disease-causing variant already identified 29 % (26/91) of the patients have a VUS of which the majority are located in MMR genes, in concordance also with other reports [6]. A patient without identified mutation in this study could have mutations in high penetrant recently identified genes, which were not included in this panel. Several candidate genes for both polyposis and non-polyposis syndromes have been identified [5, 11]. Multigene panels used for detection of pathogenic variants in CRC syndromes frequently include genes for which the cancer risk is not well known and management guidelines are not yet established. Classifying the genes into different categories based on these issues might be advisable [7]. The implementation of multigene-panel based technology into the clinic implies new opportunities and challenges which might also require introduction of new models for genetic counselling.

## Electronic supplementary material

Below is the link to the electronic supplementary material.

Supplementary material 1 (PDF 161 kb)

## References

[1] Abecasis GR, Auton A, Brooks LD, DePristo MA, Durbin RM, Handsaker RE, Kang HM, Marth GT, McVean GA (2012). An integrated map of genetic variation from 1,092 human genomes. Nature.

[2] Adam R, Spier I, Zhao B, Kloth M, Marquez J, Hinrichsen I, Kirfel J, Tafazzoli A, Horpaopan S, Uhlhaas S, Stienen D, Friedrichs N, Altmuller J, Laner A, Holzapfel S, Peters S, Kayser K, Thiele H, Holinski-Feder E, Marra G, Kristiansen G, Nothen MM, Buttner R, Moslein G, Betz RC, Brieger A, Lifton RP, Aretz S (2016). Exome sequencing identifies biallelic MSH3 germline mutations as a recessive subtype of colorectal adenomatous polyposis. Am J Hum Genet.

[3] Adzhubei I, Jordan DM, Sunyaev SR (2013). Predicting functional effect of human missense mutations using PolyPhen-2. Curr Protoc Hum Genet Chapter.

[4] Cheah PY, Wong YH, Chau YP, Loi C, Lim KH, Lim JF, Koh PK, Eu KW (2009). Germline bone morphogenesis protein receptor 1A mutation causes colorectal tumorigenesis in hereditary mixed polyposis syndrome. Am J Gastroenterol.

[5] Chubb D, Broderick P (2016). Rare disruptive mutations and their contribution to the heritable risk of colorectal cancer. Nat Commun.

[6] Cragun D, Radford C, Dolinsky JS, Caldwell M, Chao E, Pal T (2014). Panel-based testing for inherited colorectal cancer: a descriptive study of clinical testing performed by a US laboratory. Clin Genet.

[7] Fecteau H, Vogel KJ, Hanson K, Morrill-Cornelius S (2014). The evolution of cancer risk assessment in the era of next generation sequencing. J Genet Couns.

[8] Fernandez-Rozadilla C, Brea-Fernandez A, Bessa X, Alvarez-Urturi C, Abuli A, Clofent J, Paya A, Jover R, Xicola R, Llor X, Andreu M, Castells A, Carracedo A, Castellvi-Bel S, Ruiz-Ponte C (2013). BMPR1A mutations in early-onset colorectal cancer with mismatch repair proficiency. Clin Genet.

[9] Gallego CJ, Shirts BH, Bennette CS, Guzauskas G, Amendola LM, Horike-Pyne M, Hisama FM, Pritchard CC, Grady WM, Burke W, Jarvik GP, Veenstra DL (2015). Next-generation sequencing panels for the diagnosis of colorectal cancer and polyposis syndromes: a cost-effectiveness analysis. J Clin Oncol.

[10] Grandval P, Blayau M, Buisine MP, Coulet F, Maugard C, Pinson S, Remenieras A, Tinat J, Uhrhammer N, Beroud C, Olschwang S (2014). The UMD-APC database, a model of nation-wide knowledge base: update with data from 3,581 variations. Hum Mutat.

[11] Hahn MM, de Voer RM, Hoogerbrugge N, Ligtenberg MJ, Kuiper RP, van Kessel AG (2016) The genetic heterogeneity of colorectal cancer predisposition—guidelines for gene discovery. Cell Oncol. doi:10.1007/s13402-016-0284-6 10.1007/s13402-016-0284-6 PMC5121185 27279102

[12] Jaeger E, Leedham S, Lewis A, Segditsas S, Becker M, Cuadrado PR, Davis H, Kaur K, Heinimann K, Howarth K, East J, Taylor J, Thomas H, Tomlinson I (2012). Hereditary mixed polyposis syndrome is caused by a 40-kb upstream duplication that leads to increased and ectopic expression of the BMP antagonist GREM1. Nat Genet.

[13] Jass JR (2007). Gastrointestinal polyposes: clinical, pathological and molecular features. Gastroenterol Clin North Am.

[14] Kanter-Smoler G, Fritzell K, Rohlin A, Engwall Y, Hallberg B, Bergman A, Meuller J, Gronberg H, Karlsson P, Bjork J, Nordling M (2008). Clinical characterization and the mutation spectrum in Swedish adenomatous polyposis families. BMC Med.

[15] Kim IJ, Park JH, Kang HC, Kim KH, Kim JH, Ku JL, Kang SB, Park SY, Lee JS, Park JG (2003). Identification of a novel BMPR1A germline mutation in a Korean juvenile polyposis patient without SMAD4 mutation. Clin Genet.

[16] Kircher M, Witten DM, Jain P, O’Roak BJ, Cooper GM (2014). A general framework for estimating the relative pathogenicity of human genetic variants. Nat Genet.

[17] Korhonen MK, Vuorenmaa E, Nystrom M (2008). The first functional study of MLH3 mutations found in cancer patients. Genes Chromosomes Cancer.

[18] Kraus C, Rau TT, Lux P, Erlenbach-Wunsch K, Lohr S, Krumbiegel M, Thiel CT, Stohr R, Agaimy A, Croner RS, Sturzl M, Hohenberger W, Hartmann A, Reis A (2015). Comprehensive screening for mutations associated with colorectal cancer in unselected cases reveals penetrant and nonpenetrant mutations. Int J Cancer.

[19] Kravochuck SE, Church JM (2016). Hereditary non-polyposis colorectal cancer/Lynch syndrome in three dimensions. ANZ J Surg..

[20] Kumar P, Henikoff S, Ng PC (2009). Predicting the effects of coding non-synonymous variants on protein function using the SIFT algorithm. Nat Protoc.

[21] Lammi L, Arte S, Somer M, Jarvinen H, Lahermo P, Thesleff I, Pirinen S, Nieminen P (2004). Mutations in AXIN2 cause familial tooth agenesis and predispose to colorectal cancer. Am J Hum Genet.

[22] Landrum MJ, Lee JM, Riley GR, Jang W, Rubinstein WS, Church DM, Maglott DR (2014). ClinVar: public archive of relationships among sequence variation and human phenotype. Nucleic Acids Res.

[23] Lin Y, Lin S, Baxter MD, Lin L, Kennedy SM, Zhang Z, Goodfellow PJ, Chapman WC, Davidson NO (2015). Novel APC promoter and exon 1B deletion and allelic silencing in three mutation-negative classic familial adenomatous polyposis families. Genome Med.

[24] Liu C, Wang QS, Wang YJ (2012). The CHEK2 I157T variant and colorectal cancer susceptibility: a systematic review and meta-analysis. Asian Pac J Cancer Prev.

[25] Liu H, Ding T, Zhan Y, Feng H (2015). A Novel AXIN2 Missense Mutation Is Associated with Non-Syndromic Oligodontia. Plos One.

[26] Lucci-Cordisco E, Risio M, Venesio T, Genuardi M (2013). The growing complexity of the intestinal polyposis syndromes. Am J Med Genet A.

[27] McKenna A, Hanna M, Banks E, Sivachenko A, Cibulskis K, Kernytsky A, Garimella K, Altshuler D, Gabriel S, Daly M, DePristo MA (2010). The genome analysis toolkit: a mapreduce framework for analyzing next-generation DNA sequencing data. Genome Res.

[28] Neklason DW, Done MW, Sargent NR, Schwartz AG, Anton-Culver H, Griffin CA, Ahnen DJ, Schildkraut JM, Tomlinson GE, Strong LC, Miller AR, Stopfer JE, Burt RW (2011). Activating mutation in MET oncogene in familial colorectal cancer. BMC Cancer.

[29] Nieminen TT, Abdel-Rahman WM, Ristimaki A, Lappalainen M, Lahermo P, Mecklin JP, Jarvinen HJ, Peltomaki P (2011). BMPR1A mutations in hereditary nonpolyposis colorectal cancer without mismatch repair deficiency. Gastroenterology.

[30] O’Riordan JM, O’Donoghue D, Green A, Keegan D, Hawkes LA, Payne SJ, Sheahan K, Winter DC (2010). Hereditary mixed polyposis syndrome due to a BMPR1A mutation. Colorectal Dis.

[31] Palles C, Cazier JB, Howarth KM, Domingo E, Jones AM, Broderick P, Kemp Z, Spain SL, Guarino E, Salguero I, Sherborne A, Chubb D, Carvajal-Carmona LG, Ma Y, Kaur K, Dobbins S, Barclay E, Gorman M, Martin L, Kovac MB, Humphray S, Lucassen A, Holmes CC, Bentley D, Donnelly P, Taylor J, Petridis C, Roylance R, Sawyer EJ, Kerr DJ, Clark S, Grimes J, Kearsey SE, Thomas HJ, McVean G, Houlston RS, Tomlinson I (2013). Germline mutations affecting the proofreading domains of POLE and POLD1 predispose to colorectal adenomas and carcinomas. Nat Genet.

[32] Pavicic W, Nieminen TT, Gylling A, Pursiheimo JP, Laiho A, Gyenesei A, Jarvinen HJ, Peltomaki P (2014). Promoter-specific alterations of APC are a rare cause for mutation-negative familial adenomatous polyposis. Genes Chromosomes Cancer.

[33] Robinson JT, Thorvaldsdottir H, Winckler W, Guttman M, Lander ES, Getz G, Mesirov JP (2011). Integrative genomics viewer. Nat Biotech.

[34] Rohlin A, Eiengard F, Lundstam U, Zagoras T, Nilsson S, Edsjo A, Pedersen J, Svensson J, Skullman S, Karlsson BG, Bjork J, Nordling M (2016). GREM1 and POLE variants in hereditary colorectal cancer syndromes. Genes Chromosomes Cancer.

[35] Rohlin A, Engwall Y, Fritzell K, Goransson K, Bergsten A, Einbeigi Z, Nilbert M, Karlsson P, Bjork J, Nordling M (2011). Inactivation of promoter 1B of APC causes partial gene silencing: evidence for a significant role of the promoter in regulation and causative of familial adenomatous polyposis. Oncogene.

[36] Rohlin A, Zagoras T, Nilsson S, Lundstam U, Wahlstrom J, Hulten L, Martinsson T, Karlsson GB, Nordling M (2014). A mutation in POLE predisposing to a multi-tumour phenotype. Int J Oncol.

[37] Samadder NJ, Jasperson K, Burt RW (2015). Hereditary and common familial colorectal cancer: evidence for colorectal screening. Dig Dis Sci.

[38] Seifert M, Reichrath J (2006). The role of the human DNA mismatch repair gene hMSH2 in DNA repair, cell cycle control and apoptosis: implications for pathogenesis, progression and therapy of cancer. J Mol Histol.

[39] Shirts BH, Casadei S, Jacobson AL, Lee MK, Gulsuner S, Bennett RL, Miller M, Hall SA, Hampel H, Hisama FM, Naylor LV, Goetsch C, Leppig K, Tait JF, Scroggins SM, Turner EH, Livingston R, Salipante SJ, King M-C, Walsh T, Pritchard CC (2016) Improving performance of multigene panels for genomic analysis of cancer predisposition. Genet Med. doi:10.1038/gim.2015.212 10.1038/gim.2015.212 26845104

[40] Simbolo M, Mafficini A, Agostini M, Pedrazzani C, Bedin C, Urso ED, Nitti D, Turri G, Scardoni M, Fassan M, Scarpa A (2015). Next-generation sequencing for genetic testing of familial colorectal cancer syndromes. Hered Cancer Clin Pract.

[41] Snow AK, Tuohy TM, Sargent NR, Smith LJ, Burt RW, Neklason DW (2015). APC promoter 1B deletion in seven American families with familial adenomatous polyposis. Clin Genet.

[42] Stenson PD, Mort M, Ball EV, Shaw K, Phillips A, Cooper DN (2014). The human gene mutation database: building a comprehensive mutation repository for clinical and molecular genetics, diagnostic testing and personalized genomic medicine. Hum Genet.

[43] Stoffel EM, Eng C (2014). Exome sequencing in familial colorectal cancer: searching for needles in haystacks. Gastroenterology.

[44] Suchy J, Cybulski C, Wokolorczyk D, Oszurek O, Gorski B, Debniak T, Jakubowska A, Gronwald J, Huzarski T, Byrski T, Dziuba I, Gogacz M, Wisniowski R, Wandzel P, Banaszkiewicz Z, Kurzawski G, Kladny J, Narod SA, Lubinski J (2010). CHEK2 mutations and HNPCC-related colorectal cancer. Int J Cancer.

[45] Susswein LR, Marshall ML, Nusbaum R, Vogel Postula KJ, Weissman SM, Yackowski L, Vaccari EM, Bissonnette J, Booker JK, Cremona ML, Gibellini F, Murphy PD, Pineda-Alvarez DE, Pollevick GD, Xu Z, Richard G, Bale S, Klein RT, Hruska KS, Chung WK (2015) Pathogenic and likely pathogenic variant prevalence among the first 10,000 patients referred for next-generation cancer panel testing. Genet Med. doi:10.1038/gim.2015.166 10.1038/gim.2015.166 PMC4985612 26681312

[46] Thompson BA, Spurdle AB, Plazzer JP, Greenblatt MS, Akagi K, Al-Mulla F, Bapat B, Bernstein I, Capella G, den Dunnen JT, du Sart D, Fabre A, Farrell MP, Farrington SM, Frayling IM, Frebourg T, Goldgar DE, Heinen CD, Holinski-Feder E, Kohonen-Corish M, Robinson KL, Leung SY, Martins A, Moller P, Morak M, Nystrom M, Peltomaki P, Pineda M, Qi M, Ramesar R, Rasmussen LJ, Royer-Pokora B, Scott RJ, Sijmons R, Tavtigian SV, Tops CM, Weber T, Wijnen J, Woods MO, Macrae F, Genuardi M (2014). Application of a 5-tiered scheme for standardized classification of 2,360 unique mismatch repair gene variants in the InSiGHT locus-specific database. Nat Genet.

[47] Tomlinson I, Jaeger E, Leedham S, Thomas H (2013). Reply to “The classification of intestinal polyposis”. Nat Genet.

[48] Valle L (2014). Genetic predisposition to colorectal cancer: where we stand and future perspectives. World J Gastroenterol.

[49] Valle L, Hernandez-Illan E, Bellido F, Aiza G, Castillejo A, Castillejo MI, Navarro M, Segui N, Vargas G, Guarinos C, Juarez M, Sanjuan X, Iglesias S, Alenda C, Egoavil C, Segura A, Juan MJ, Rodriguez-Soler M, Brunet J, Gonzalez S, Jover R, Lazaro C, Capella G, Pineda M, Soto JL, Blanco I (2014). New insights into POLE and POLD1 germline mutations in familial colorectal cancer and polyposis. Hum Mol Genet.

[50] Weren RD, Ligtenberg MJ, Kets CM, de Voer RM, Verwiel ET, Spruijt L, van Zelst-Stams WA, Jongmans MC, Gilissen C (2015). A germline homozygous mutation in the base-excision repair gene NTHL1 causes adenomatous polyposis and colorectal cancer. Nat Genet.

[51] Whitelaw SC, Murday VA, Tomlinson IP, Thomas HJ, Cottrell S, Ginsberg A, Bukofzer S, Hodgson SV, Skudowitz RB, Jass JR, Talbot IC, Northover JM, Bodmer WF, Solomon E (1997). Clinical and molecular features of the hereditary mixed polyposis syndrome. Gastroenterology.

[52] Wong S, Liu H, Bai B, Chang H, Zhao H, Wang Y, Han D, Feng H (2014). Novel missense mutations in the AXIN2 gene associated with non-syndromic oligodontia. Arch Oral Biol.

[53] Xiang HP, Geng XP, Ge WW, Li H (2011). Meta-analysis of CHEK2 1100delC variant and colorectal cancer susceptibility. Eur J Cancer.

[54] Yurgelun MB, Allen B, Kaldate RR, Bowles KR, Judkins T, Kaushik P, Roa BB, Wenstrup RJ, Hartman AR, Syngal S (2015). Identification of a Variety of Mutations in Cancer Predisposition Genes in Patients With Suspected Lynch Syndrome. Gastroenterology.

