# Supplementary material for: Expanding the genotype–phenotype spectrum in hereditary colorectal cancer by gene panel testing
Source: Fam Cancer. 2016 Sep 30;16(2):195–203. doi: 10.1007/s10689-016-9934-0 (PMC5357488; doi:10.1007/s10689-016-9934-0)
Supplement: Supplementary file 1 — Supplementary material 1 (PDF 161 kb) [file 10689_2016_9934_MOESM1_ESM.pdf]

Supplementary Table 1 Click here to download attachment to manuscript Supplementary Table 1_160825 .docx
Click here to view linked References

Supplementary Table 1 Clinical characteristics of patients in group I to VI

Patient no	Gender	Age at diagnosis	MSI	IHC loss	Am criteria	No of polyps	CRC	Location	Stage	Other
**I) CRC familial or unknown inheritance not polyposis**										
1	f	75	MSS	–	yes	–	yes	sigmoid	–	
2	f	26	MSS	–	no	–	yes	cecum	II	
3	m	60	MSS	–	no	–	yes	sigmoid	III	
4	m	27	MSS	–	yes	–	yes	–	II	ulcerative colitis at 10 years
5	f	41	MSS	–	yes	–	yes	sigmoid	II	
6	f	41	MSS	–	no	–	yes	–	II	breast cancer
7	f	35	MSS	–	no		yes	rectum	III	
8	f	37	MSS	–	no	–	yes	rectum	III	
9	f	62	MSS	–	no	–	yes	appendix	–	
10	f	58	MSS	–	yes	–	yes	rectum	–	
11	f	42	MSS	–	yes	–	yes	rectum	–	
12	f	–	MSS	–	yes	–	yes	rectum	–	
13	f	33	MSI low	–	no	–	yes	–	–	
14	f	51	MSS	–	no	–	–	sigmoid	–	breast cancer
15	f	52	MSS	–	no	1-100	–	–	–	endometrial cancer
16	f	82	MSS	–	yes	–	–	cecum	–	
17	f	46	MSS	–	yes	–	yes	rectum	III	
18	f	53	MSS	–	no	1-100	yes	–	III	
19	f	40	MSS	–	yes	1-100	yes	–	III	
20	m	76	MSS	–	no	1-100	yes	sigmoid	–	
21	f	38	MSS	–	no	1-100	yes	sigmoid	II	
22	m	74	MSS	–	yes	–	yes	rectum	–	
23	m	48	MSS	–	yes	–	yes	cecum	II	

24	m	60	MSS	–	yes	1-100	yes	sigmoid	–	
25	f	51	MSS	–	yes	1-100	yes	–	I	
26	f	50	MSI-H	–	no	–	yes	–	–	endometrial cancer
27	f	40	MSI-H	–	no	1-100	yes	ascending	II	sigmoid polyps
28	m	41	MSS	–	no	–	yes	transverse	–	
29	m	31	MSS	–	no	–	–	–	–	
30	f	32	MSS	–	no	–	yes	–	–	
31	f	23	MSS	–	no	–	yes	sigmoid	III	
32	f	31	MSS	–	yes	–	yes	–	–	
33	f	51	MSS	–	–	–	yes	–	–	
34	f	66	MSI-H	MLH1/PMS2	no	–	yes	caecum	II	
35	f	61	MSI-H	MLH1/PMS2	no	–	yes	caecum	II	
36	m	51	MSI-H	MLH1/PMS2	no	–	yes	rectum	II	rectal cancer
37	f	70	MSI-H	MLH1/PMS2	no	–	yes	transverse	II	endometrial cancer
38	f	63	MSI-H	MLH1/PMS2	no	–	no	–	I	endometrial cancer
39	m	49	MSI-H	MLH1/PMS2	no	–	yes	ascendens	II	
40	f	61	MSI-H	–	no	–	yes	ascendens	II	
41	f	57	MSI-H	MLH1/PMS2	yes	–	yes	ascendens	III	
42	f	50	MSI-H	MLH1/PMS2	no	–	yes	ascendens	II	
43	f	41	MSI-H	MLH1/PMS2	no	–	yes	transverse	II	
44	f	38	MSI-H	MLH1/PMS2	no	–	yes	ascendens	IV	breast cancer, 32 years
45	f	40	MSI-H	MLH1/PMS2	yes	–	no	–	I	endometrial cancer
46	f	57	MSI-H	MLH1/PMS2	no	–	no	–	I	endometrial cancer
47	f	68	MSI-H	MLH1/PMS2	no	–	yes	ascendens	II	endometrial cancer, 53 years
48	f	55	MSI-H	MSH6	no	–	yes	ascendens	II	synchronous endometrial cancer, 55 years
49	f	21	MSI-H	MSH6	no	–	yes	rectum	III	childhood brain tumour
50	f	80	MSI-H	MSH6	yes	–	yes	ascendens	II	endometrial cancer, 50 years

51	f	35	MSI-H	–	no	–	yes	rectum	III	
52	m	76	MSS	–	yes	–	yes	ascendens	II	
54	f	48	MSI-H	–	no	–	–	–	I	endometrial cancer
55	f	79	MSS	–	no	–	yes	rectum	II	rectal cancer
56	m	54	MSI-H	–	yes	–	yes	rectum	I	rectal cancer
57	m	41	MSI-H	–	no	–	yes	ascendens	III	
91	m	39	MSI-H	–	yes	–	yes	–	–	
92	f	45	MSI-H	–	no	–	yes	–	–	endometrial cancer, 45 years, breast cancer, 53 years
**II) Unexplained adenomatous polyposis >100 polyps, inheritance**										
58	f	17	–	–	–	>1000	no	–	–	
59	f	31	–	–	–	101-1000	yes	descending	–	several family members with polyps and crc
**III) Unexplained adenomatous polyposis 1-100 polyps, inheritance**										
60	–	21	–	–	–	1-100	–	–	–	
61	m	–	–	–	–	–	–	–	–	
62	f	38	–	–	–	1-100	–	descending	–	
63	f	45	MSS	–	–	1-100	–	sigmoid	–	
64	m	35	–	–	–	1-100	–	–	–	tubular polyps
65	m	66	–	–	–	1-100?	yes	–	–	
66	f	41	–	–	–	1-100	yes	rectum	–	sister of the paternal grandmother with breast cancer, 31 years
67	f	41	–	–	–	1-100	yes	–	–	
**IV) Unexplained adenomatous polyposis unknown inheritance**										
68	f	84	–	–	–	1-100	–	–	–	
69	m	48	–	–	–	1-100	yes	rectum	–	duodenal polyp
70	m	57	–	–	–	1-100	yes	descending	–	FGP
71	f	47	–	–	–	1-100	yes	sigmoid (x2)	–	flat adenomas
72	m	31	–	–	–	1-100	no	–	–	FGP

73	m	58	–	–	–	1-100	no	–	–	
74	f	46	–	–	–	1-100	yes	–	–	
75	m	60	–	–	–	1-100	yes	sigmoid	–	
76	f	52	–	–	–	1-100	yes	descending	–	
**V) Familial or simplex atypical polyposis/mixed polyposis/serrated polyposis**										
77	f	60	–	–	–	1-100	yes	rectum	–	atypical polyposis
78	f	50	–	–	–	1-1000	yes	rectum	–	
79	f	50	–	–	–	1-100	yes	descending (x3)	–	
80	f	51	–	–	–	1-100	no	–	–	duodenal polyps
81	f	11	–	–	–	1-100	–	–	–	no inheritance
82	m	45	MSS	–	no	1-100	–	–	–	
83	f	54	–	–	–	1-100	yes	–	–	breast cancer, 59 years
84	f	36	–	–	–	100-200	–	–	–	mixed polyposis
85	m	71	–	–	–	1-100	–	–	–	several family members and both parents with crc
86	m	74	–	–	–	1-100	–	–	–	
87	m	<50	–	–	–	1-100	–	–	–	mixed polyposis
88	f	–	–	–	–	1-100	–	–	–	mixed polyposis
**VI) Polymerase proofreading associated polyposis (PPAP)**										
89	f	–	MSS	–	yes	1-100	yes	–	–	See Rohlin et al 2014
90	m	43	MSS	–	no	–	yes	sigmoid	III	See Rohlin et al 2016
neg-negative, Am-Amsterdam, FGP-fundic gland polyps, IHC-immunohistochemistry, MSS-microsatellite stable, MSI-microsatellite instable										
